# A DAMP-Based Assay for Rapid and Affordable Diagnosis of Bacterial Meningitis Agents: *Haemophilus influenzae*, *Neisseria meningitidis*, and *Streptococcus pneumoniae*

**DOI:** 10.3390/ijms25158282

**Published:** 2024-07-29

**Authors:** Liubov A. Shkodenko, Al-Abbass Mohamed, Muhannad Ateiah, Maria S. Rubel, Elena I. Koshel

**Affiliations:** Laboratory of DNA-Nanosensor Diagnostics, ITMO University, Lomonosova Street, 9, 191002 St Petersburg, Russia; shkodenko@scamt-itmo.ru (L.A.S.); alabbass@scamt-itmo.ru (A.-A.M.); muhannad@scamt-itmo.ru (M.A.); msrubel@itmo.ru (M.S.R.)

**Keywords:** isotheral amplification, DAMP, CNS infections, meningitis, DNA diagnostics, POC, diagnostic assay

## Abstract

The rapid and accurate diagnosis of meningitis is critical for preventing severe complications and fatalities. This study addresses the need for accessible diagnostics in the absence of specialized equipment by developing a novel diagnostic assay. The assay utilizes dual-priming isothermal amplification (DAMP) with unique internal primers to significantly reduce non-specificity. For fluorescence detection, the dye was selected among Brilliant Green, Thioflavin T, and dsGreen. Brilliant Green is preferred for this assay due to its availability, high fluorescence level, and optimal sample-to-background (S/B) ratio. The assay was developed for the detection of the primary causative agents of meningitis (*Haemophilus influenzae*, *Neisseria meningitidis*, and *Streptococcus pneumoniae*), and tested on clinical samples. The developed method demonstrated high specificity, no false positives, sensitivity comparable to that of loop-mediated isothermal amplification (LAMP), and a high S/B ratio. This versatile assay can be utilized as a standalone test or an integrated assay into point-of-care systems for rapid and reliable pathogen detection.

## 1. Introduction

Fast and accessible diagnosis of infectious diseases plays a critical role in facilitating effective therapy. This holds particular significance in the context of infectious brain inflammations, such as meningitis and encephalitis, where timely administration of the appropriate treatment is paramount to preventing disabling complications and mortality [1]. To achieve this, the diagnostic approach must be sensitive, specific, and accessible. Within this context, nucleic acid amplification testing (NAAT) methods are the golden standard of molecular diagnostics [2,3]. Among the NAAT methods, PCR-based technologies and DNA microarray assays are widely used in clinical practice, with numerous approaches developed [4]. Evidence suggests that NAATs-based methods remain a crucial diagnostic tool [5], especially when CSF cultures yield negative results [6] as these culturing methods become significantly limited upon antibiotic treatment [7].

Among NAAT methods, isothermal amplification is usually well suited for any application, especially for Point-of-care (POC) systems [8]. These methods eliminate the need for laboratory equipment that is essential in conventional PCR-based diagnostics [4,9]. Loop-mediated isothermal amplification (LAMP) stands out as the most prevalent technique in commercial settings [10,11]. LAMP has gained prominence due to its simplicity, rapidity, and high sensitivity [12]. These features make LAMP a suitable method for primary screening interventions. However, it has inherent limitations, mainly the potential for non-specific amplification, which often entails an additional PCR test to confirm the true positivity of results [13,14]. Therefore, despite the various advantages of LAMP and given that it is the most common method of isothermal amplification, non-specific reactions remain its biggest drawback [15].

A novel method of isothermal amplification, known as dual priming isothermal amplification (DAMP) has been introduced to address this issue [16]. This approach utilizes the concept of a “dual-priming” strand extension mechanism through incorporating two pairing-competition primers and designing unique inner primers, thereby enabling highly sensitive nucleic acid detection with increasingly low non-specific products. Based on this feature, DAMP represents a promising approach for the development of rapid and efficient diagnostic methods suited for meningitis detection, whether within traditional laboratory settings or adaptable for POC systems.

The detection of the amplification product is an essential aspect that must be considered when developing any NAAT-based method. There are two primary strategies for detecting isothermal amplification products, direct methods using DNA intercalating fluorophores [12] and indirect methods such as colorimetric detection, which rely on changes in the medium’s composition rather than nucleic acid concentration [17]. Direct fluorescent detection provides quantitative information about the reaction, as well as high sensitivity towards the amplification product [18].

In pursuit of achieving the most precise fluorescence-based detection of double-stranded DNA (dsDNA), dyes with the highest sample-to-background ratio (S/B ratio) were carefully selected. This parameter holds significance in mitigating additional non-specific background signals during the detection of isothermal amplification products [19,20]. This study focused on evaluating three primary dyes: the standard dye dsGreen, commonly utilized for assessing non-specific amplification in LAMP reactions [14]; Thioflavin T, known for its efficient selective binding to dsDNA apart from ssDNA [21]; and Brilliant Green, recognized for its affordability and purportedly high sensitivity in dsDNA detection [22].

Our research has primarily targeted towards the detection of the three predominant bacterial pathogens associated with brain inflammation, namely, *Haemophilus influenzae*, *Neisseria meningitidis*, and *Streptococcus pneumoniae*. These pathogens are identified as the leading causing agents of meningitis in individuals beyond the neonatal period [1,23,24]. Consequently, the investigation of these three specific pathogens within the context of diagnostics has been a focal point of our studies [25,26,27,28,29,30] and in the work of others [31,32,33].

Herein, we report a highly sensitive and selective assay for the detection of these three aforementioned pathogens. The assay employs a DAMP reaction featuring three distinct fluorescent dyes (dsGreen, Thioflavin T, and Brilliant Green) for visualization of the amplification products. The assay also underwent evaluation using clinical samples of cerebrospinal fluid (CSF). This assay stands out for its versatility, being suitable for standalone use or for integration within POC devices owing to its simplicity and reliability.

## 2. Results

### 2.1. DAMP Primers Design

Compared to the traditional approach of LAMP assays that used six primers targeting eight distinct sites, DAMP utilizes six primers that recognize six distinct sites, thereby simplifying the complexity of primer design and reducing the potential risk of having non-specific reactions.

The primers were designed to target specific genes that are distinct for each pathogen. For *H. influenzae*, the target gene is *hpd* (accession: MN488730) [34]. For *N. meningitidis*, the target gene is *ctrA* (accession: NZ_CP021520) [35]. As for *S. pneumoniae*, the autolytic gene *lytA* (accession: AE005672) is a staple in nucleic acid detection [36]. These targets were selected as they are highly conserved among various strain of their respective species. Designed primers can be found in Table 1.

### 2.2. Limit of Detection

**Figure 1:**
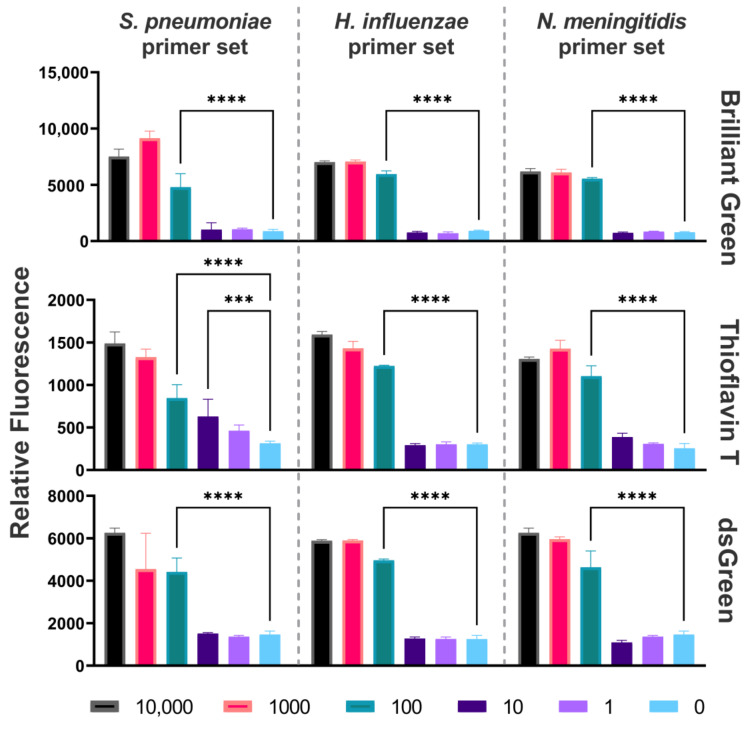
Sensitivity of DAMP assay. Relative fluorescence of the DAMP products for *N. meningitidis*, *S. pneumoniae*, and *H. influenzae* with different Ges, measured using Brilliant Green, Thioflavin T, and dsGreen. *** *p* < 0.001; **** *p* < 0.0001.

To assess the sensitivity of the DAMP assay, tenfold serial dilutions of *N. meningitidis*, *S. pneumoniae* and *H. influenzae* DNA from 10,000 to 1 GE were performed. All diluted DNA samples were amplified by DAMP reaction. The sensitivity limit was reached at 100 GE. Detection of amplification products was carried out by measuring the relative fluorescence with the addition of dyes (Figure 1), and by gel electrophoresis for validation (Figure S1).

The fluorescence signal of the amplification products was detected using three distinct dyes: dsGreen, Thioflavin T, and Brilliant Green. The sample-to-background ratio exceeded the established threshold, where the reaction with 0 GE of DNA was considered the background. The S/B ratios for each primer set with their respective samples for each dye can be seen in Table S1. The S/B ratio of Brilliant Green was higher than that of Thioflavin T and dsGreen by an average of 41% and 48%, respectively, demonstrating greater efficiency for the detection of amplification products.

### 2.3. Specificity

**Figure 2:**
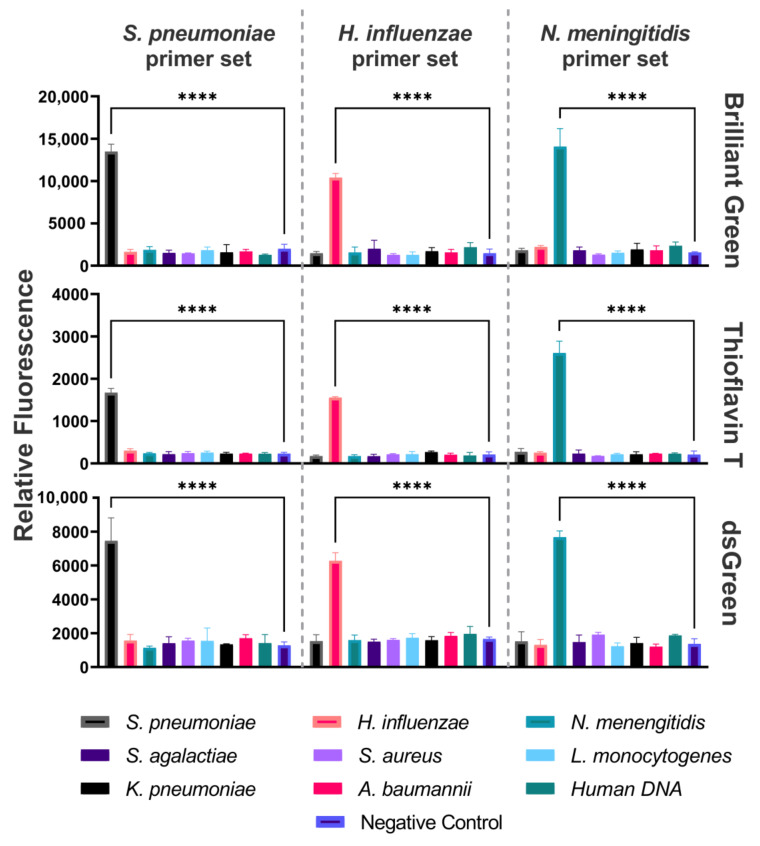
Specificity of DAMP assay. Relative fluorescence of the DAMP amplification product for each of the three primer sets using Brilliant Green, Thioflavin T, and dsGreen. **** *p* < 0.0001.

To assess the specificity of DAMP reactions, a cross-reaction assay was performed on *N. meningitidis*, *S. pneumoniae*, and *H. influenzae*, the primary etiological agents of bacterial meningitis, and on *Streptococcus agalactiae*, *Staphylococcus aureus*, *Listeria monocytogenes*, *Klebsiella pneumoniae*, *Acinetobacter baumannii*, as well as against fragmented human DNA (Evrogen, Moscow, Russia) representative of the host genetic material. The experimental findings from the DAMP specificity test unequivocally demonstrated the absence of any discernible cross-reactivity among the targeted DNA fragments, as they were detected by measuring the relative fluorescence with the addition of dyes (Figure 2). The reactions were validated with agarose gel electrophoresis (Figure S2). Fluorescence detection with the three dyes confirmed the absence of non-specificity. When the introduced DNA failed to match the primer sequences, the fluorescence intensity was at the background level.

### 2.4. Clinical Samples Testing

**Figure 3:**
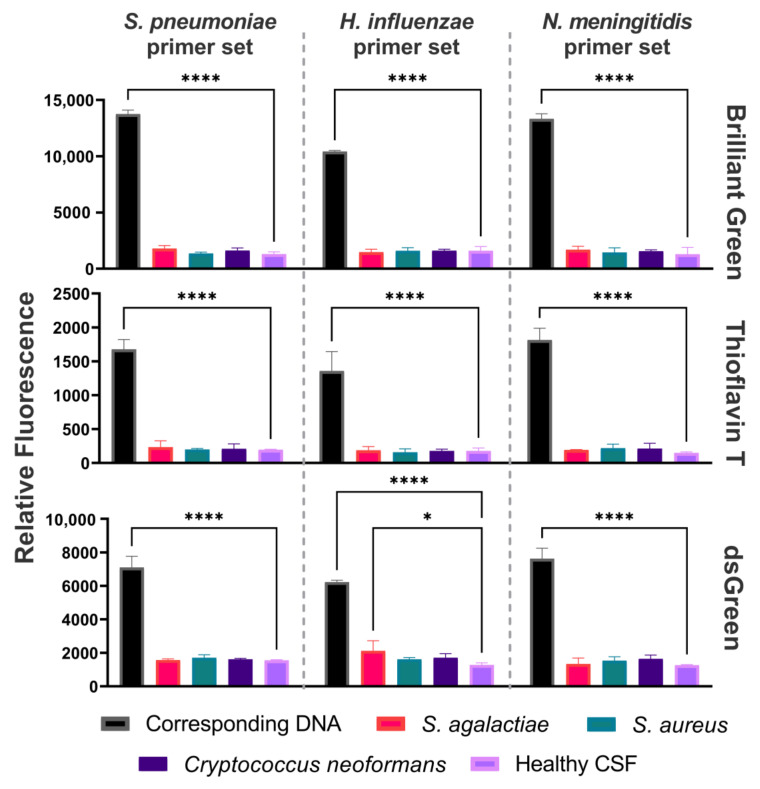
Testing of the CSF clinical samples by DAMP assay. Fluorescence detection of DAMP amplicons using three primer sets on samples containing one of the pathogens: *N. meningitidis*, *S. pneumoniae*, *H. influenzae*, *S. agalactiae*, *S. aureus*, or *C. neoformans*. Relative fluorescence for each of the three primer sets using Brilliant Green, Thioflavin T, and dsGreen. * *p* < 0.05, **** *p* < 0.0001.

DAMP amplification was performed on DNA isolated from cerebrospinal fluid (CSF) samples. Each primer set was tested on clinical samples containing one of the pathogens: *N. meningitidis*, *S. pneumoniae*, *H. influenzae*, *S. agalactiae*, *S. aureus*, or *Cryptococcus neoformans*, with an extra healthy CSF sample that was used as the negative control to test for background signals (Figure 3). Fluorescence detection of the amplification products derived from CSF clinical samples was conducted utilizing three dyes: Brilliant Green, Thioflavin T, and dsGreen. The reactions were validated with agarose gel electrophoresis (Figure S3).

## 3. Discussion

Early detection through POC systems can mitigate the risk of neurological damage, enhance patient outcomes, and support prompt clinical decision-making, particularly in critical scenarios requiring immediate intervention [37]. The current methods are carried out by cultivating a CSF culture and waiting for its growth results, which can take up to 2 days [38]. Guidelines recommend decreasing the time between admission and treatment as much as possible to reduce the mortality rate, patient recovery outcomes, reduce healthcare costs, and enhance overall public health [39]. To achieve this aim, a testing system that integrates nucleic acid extraction with amplification and detection is required [40,41]. However, it has been proven challenging to combine all the previous aspects within one testing system [42]. The method described herein can be easily integrated into such an automated system and provide a solution to the challenges described above.

NAAT-based methods have proven to be effective in providing secondary confirmation to CSF culture results, but not as a primary testing method [5]. Isothermal amplification methods a of especial interest due to their speed and sensitivity, which makes them eligible to becoming primary diagnostic tools upon ideal optimization [16,43]. However, the main drawback to these methods is non-specific reactions that are mainly caused by the presence of background DNA and cross-contamination with exogenous DNA in clinical samples, including those obtained from the CNS [44]. This is especially true when dealing with methods, such as LAMP, that are prone to false positives due to the complex primer designing processes, which leads to non-specific and non-template amplification products [13].

The occurrence of false positivity can be attributed to primer dimer formation, as well as to cross contamination [15]. Dimers formation is a consequence of the LAMP reactions, where multiple long and highly similar primers are used to amplify the target DNA [45]. Cross-contamination is a consequence of the relatively high sensitivity of LAMP, as small aerosol droplets or via carryover [46]. Some strategies have been developed to counter dimer formation and cross-contamination issues [47,48,49]. However, these strategies are not effective against high concentrations of carryover contamination [50], which is often the case in POC settings were many clinical samples are tested simultaneously and the testing system is prone to contamination by exogenous DNA. In a comparative review using COVID-19 POC systems as a model, Kang et al. explored the currently available methods of NAATs for the detection of COVID-19 viral RNA [4]. RT-LAMP provided high sensitivity and relatively fast time (15–60 min), but the false positivity was still an issue.

DAMP, the amplification method used here, is a novel approach to isothermal amplification reactions that is derived from LAMP with possibly a lower chance of false positivity [16]. The efficacy of the developed methodology was validated through the analysis of clinical specimens. The detection outcomes corresponded with the results obtained from molecular diagnostic assays routinely employed in clinical settings, including quantitative PCR (qPCR) and culturing methodologies. The method achieved a detection limit of 100 GE, which is satisfactory according to standard guidelines [1]. This limit represents a tenfold enhancement in sensitivity compared to our prior LAMP-based assay for *N. meningitidis* [25]. Nevertheless, it is still tenfold higher than the currently presented limit of detection of LAMP-based methods [43,51,52,53,54]. 

An easy and efficient detection method is paramount for a fast and accessible assay. Conventional laboratory equipment such as a fluorimeter, typically employed for monitoring nucleic acid isolation processes, suffices for endpoint detection. While colorimetric detection may require less equipment and reagents, it can be affected by the sample’s constitution, especially the pH changes [55]. This is what arguably makes fluorescence detection the ideal method for detecting POC system amplification results, as its fluorescence signal is directly affected by the nucleic acid concentrations. However, its main drawback lies in the relatively high background signal, which can reduce the reaction’s sensitivity [56].

We used three dyes to address the issue of high background signals. Among the dyes that were used to detect DAMP reaction products, Brilliant Green and Thioflavin T had comparable S/B ratios in bacterial samples, while Thioflavin T had a slightly higher ratio in clinical samples. Brilliant Green, being the cheapest and most available of the dyes, showed a high level of fluorescence in all cases. While dsGreen (which is an analogue for SYBR Green) is used as a standard dye for isothermal amplification products detection, it showed a lower S/B ratio in comparison to the other two days, even showing a tendency towards false positivity with a clinical sample (Figure 3). The amplification method in combination with the detection method used have shown a higher S/B ratio in general in comparison with a recent study by our colleagues [27]. The acquired data pertaining to dye selection may also be applicable for the detection of LAMP products. 

The identified attributes of the diagnostic assay enable its utilization as a standalone kit and its integration as a preconfigured molecular solution into a POC system. This integration is feasible due to the following reasons: (1) it requires a constant temperature for amplification, like LAMP, with a relatively enhanced protection against false positive outcomes; (2) detection can be accomplished using a basic fluorimeter; and (3) the system exhibits sensitivity comparable to that of LAMP and specificity akin to PCR.

In the prospects of the development of new diagnostic systems that require minimal time and equipment for analysis, we recommend utilizing a DAMP-based system. Apart from the mentioned advantages, DAMP may also streamline the process of selecting effective primers in a minimal timeframe. Designing LAMP primers typically requires thorough analysis and validation to create a set of primers capable of producing specific reactions devoid of false positives. Although similar to LAMP primers in many aspects, the designing process for this method achieves high specificity without yielding false positive outcomes. Remarkably, the primers formulated in this study demonstrated immediate success upon initial design, without the need for subsequent primer modifications. Primer design for other isothermal amplification methods has also proven to be difficult and often requires a few iterations before reaching a successful set of primers. Moreover, other types of isothermal amplification also exhibit the same issue when it comes to primer design. This includes, but is not limited to, helicase-dependent isothermal amplification (HDA) [57], isothermal strand displacement amplification (iSDA) [58], and recombinase polymerase amplification (RPA) [59].

## 4. Materials and Methods

### 4.1. Sample Collection and DNA Isolation

Experiments were performed on genomic DNA samples from *H. influenzae*, *N. meningitidis*, *S. pneumoniae*, and *S. agalactiae*. Thermally inactivated cell cultures of these three pathogens were obtained from the Pediatric Research and Clinical Center for Infectious Diseases (PRCCID) under the Russian Federal Medical Biological Agency. The pathogens were clinical isolates identified according to the PRCCID’s diagnostic standards. Genomic DNA was extracted from the cell cultures using the standard phenol–chloroform extraction method [60].

Thermally inactivated clinical samples of cerebrospinal fluid (CSF) were also provided by the PRCCID. These CSF samples were collected via lumbar puncture and diagnosed by qPCR AmpliSens^®^ kit (AmpliSens, Moscow, Russia) on the site of the PRCCID. Ethical consent for the usage of clinical samples for research purposes was obtained during the admission process according to the PRCCID standards and regulations. Samples were received without accompanying patient data.

### 4.2. DAMP Primer Design

When designing DAMP primers, the following criteria were considered: (1) ensuring that the spacing between the primer sections F2 and F1c (R2 and R1c) and between F3 and F2 (R3 and R2) is smaller compared to typical LAMP primers, and (2) incorporating the F1c and R1c primer sections independently into the reaction as standalone primers denoted as FC and RC, respectively. The primers FO and RO are responsible for specificity, resembling primers F3 and R3 in LAMP reactions. Furthermore, the FI and RI primers consist of the F1c + F2 and R1c + R2 primer sections, respectively, resembling the FIP and BIP primers in LAMP reactions. Primers were generated using the NEB LAMP primer design tool with modifications to parameters to make fit the DAMP working principle. The full description of the DAMP primer design can be found in the original article [16].

### 4.3. DAMP Reactions

The isothermal amplification of genomic DNA was conducted using DAMP method as described in its debut article [16]. The reaction mix contained the following reagents with their concentrations: FO and RO primers 0.2 µm each, FI and RI primers 1.6 µm each, FC and RC primers 1.6 µm each, 1.6 mm of dNTPs (Evrogen, Moscow, Russia), 6 mm of MgSO4 (Evrogen, Russia), 0.5% ethylene glycol (Lenreactiv, Saint Petersburg, Russia), 1× of Bst (*Bacillus stearothermophilus*) polymerase buffer (Genterra, Moscow, Russia), and 1.25 U of Bst Polymerase (Biolabmix, Moscow, Russia). The previous reaction mix was made, and 1 ng of extracted DNA was added. The reaction mix was then incubated at 65 °C 30–60 min. In the negative control group, water served as a substitute for nucleic acids. All experiments were performed in triplets.

For DAMP sensitivity tests, reactions were made with 10-fold dilutions of DNA amounts ranging from 10,000 to 1 genome equivalent (GE). The weight of one GE was evaluated according to the size of the reference genomes in databases and calculated as 0.0024 pg of one *N. meningitidis* GE, 0.0020 pg of one *H. influenzae* GE, and 0.0022 pg of one *S. pneumoniae* GE.

To determine the specificity of the assay, each set of primers was tested against its target pathogen and DNA of the two other pathogens from the target group. In addition to testing against DNA extracts from *Streptococcus agalactiae*, *S. aureus*, *Listeria monocytogenes*, *Klebsiella pneumoniae*, and *Acinetobacter baumannii*, as well as against fragmented human DNA (Evrogen, Russia), these bacteria were chosen based on their relative similarity to the target pathogens’ DNA [61,62,63] and the availability of clinical isolates within the partner institute of PRCCID. For specificity tests, all reactions were conducted with 1000 GE of DNA.

### 4.4. DAMP Detection

Detecting dsDNA was also performed using three different fluorescent dyes: Brilliant Green (Lenreactiv, Russia), Thioflavin T (Sigma Aldrich, St. Louis, MO, USA), and dsGreen (Lumiprobe, Moscow, Russia) with final concentrations of 30 µm, 20 µm, and 1X, respectively. Analytes and dyes were solved in nuclease-free water (Evrogen, Russia). The amplification products were added to the dyes for 5 min at room temperature, followed by mixing via vortex for 1 min. Subsequently, the fluorescent intensity was measured using a Tecan SPARK microplate reader (Switzerland) at the corresponding excitation and emission wavelengths specific to each dye [64]. The excitation and emission wavelengths were 610 and 657 nm for Brilliant Green, 442 and 487 nm for Thioflavin T, and 454 and 524 nm for dsGreen.

To validate the reactions outcome, electrophoresis was performed in a 2% agarose gel, which is a common practice in confirming the reaction results [50]. The gel electrophoresis was carried in 1× TAE buffer with ethidium bromide staining in a final concentration of 1 µg/mL. As a reference marker for fragments lengths, a 50 bp+ DNA ladder (Evrogen, Russia) was used. The visualized amplified fragments demonstrated a ladder pattern, with product lengths starting from 100 bp and above.

### 4.5. Statistical Analysis

Each experiment was carried out three times in three repetitions. The numeric data were expressed as a mean ± standard deviation. The threshold was determined as 3 standard deviations above the mean blank signal [64]. The *p*-value was calculated via GraphPad Prism 9.3.1.

## 5. Conclusions

In this study, we developed a DAMP-based approach for identifying the three primary pathogens responsible for bacterial meningitis. Our method exhibits the capability to detect bacterial DNA at concentrations as low as 100 genome copies per reaction. Moreover, it demonstrates high specificity in pathogen detection, ensuring the absence of false-positive results. This DAMP-based method can provide a rapid and accurate answer to the pathogen behind the infection for swift intervention, especially within resource-limited settings. Brilliant Green and Thioflavin T proved to be more effective for this assay as they are available in most laboratory settings and provide high fluorescence level and optimal sample-to-background (S/B) ratio. This allows for the use of fluorescence detection when using a basic fluorimeter. This opens the opportunity to incorporate the suggested method within an automated POC system for fast and reliable detection of bacterial presence. 

## Figures and Tables

**Table 1:** Primers designed for DAMP reactions.

Pathogen	Primer	Sequence (5′ ⮕ 3′)
*Streptococcus pneumoniae*	FO	ACAGGCTGGAAGAAAATCGC
	RO	GCCATCTGGCTCTACTGTGA
	FI	TGGCGCCTTCTTTAGCGTCTAATTTTCAACGAAGAAGGTGCCATGA
	RI	ATCCAGTCAGCGGACGGAACATTTTGGCTTGTCTGCCAGTGTT
	FC	TGGCGCCTTCTTTAGCGTCTAA
	RC	ATCCAGTCAGCGGACGGAACA
*Haemophilus influenzae*	FO	ACACTCTTCTGTGGACACTA
	RO	ACGATGACGATTTGGGAATT
	FI	GCAAATGCAAGCGCTTTAGACTATTATCATTGCTCACCGTGG
	RI	AGCGATGACAAAAGATGGTCGTGCGACGTCAGTTAAACCG
	FC	GCAAATGCAAGCGCTTTAGACT
	RC	AGCGATGACAAAAGATGGTCGT
*Neisseria meningitidis*	FO	AGTTGCCAGAGCAGTTGG
	RO	CGCACACTATTCCCAGCAC
	FI	GGCGTTTTACCGACCACCGATTTTTGGCACGTGGTACGGTTTC
	RI	AGGCCGCCTGAAAAAAATGGCTTTTCGACACATTCGCCGCATTA
	FC	GGCGTTTTACCGACCACCGA
	RC	AGGCCGCCTGAAAAAAATGGC

## Data Availability

The original contributions presented in the study are included in the article material; further inquiries can be directed to the corresponding author.

## Supplementary Materials

The following supporting information can be downloaded at: https://www.mdpi.com/article/10.3390/ijms25158282/s1.
