# Supplementary material for: A DAMP-Based Assay for Rapid and Affordable Diagnosis of Bacterial Meningitis Agents: Haemophilus influenzae, Neisseria meningitidis, and Streptococcus pneumoniae
Source: Int J Mol Sci. 2024 Jul 29;25(15):8282. doi: 10.3390/ijms25158282 (PMC11311791; doi:10.3390/ijms25158282)
Supplement: Supplementary file 1 [file ijms-25-08282-s001.zip › ijms-3120905-supplementary.pdf]

**Table S1.** Sample-to-background ratios of DAMP products for 10000, 1000, and 100 GE for each primer set using different dyes.

| Primer Set | Brilliant Green | | | Thioflavin T | | | dsGreen | | |
|---|---|---|---|---|---|---|---|---|---|
| | 10000 | 1000 | 100 | 10000 | 1000 | 100 | 10000 | 1000 | 100 |
| *Streptococcus Pneumoniae* | 8.49 | 10.33 | 5.42 | 4.74 | 4.23 | 2.70 | 4.27 | 3.10 | 3.01 |
| *Haemophilus influenzae* | 7.69 | 7.75 | 6.51 | 5.26 | 4.72 | 4.04 | 4.72 | 4.73 | 3.98 |
| *Neisseria meningitidis* | 7.98 | 7.86 | 7.13 | 5.14 | 5.61 | 4.34 | 4.28 | 4.06 | 3.16 |

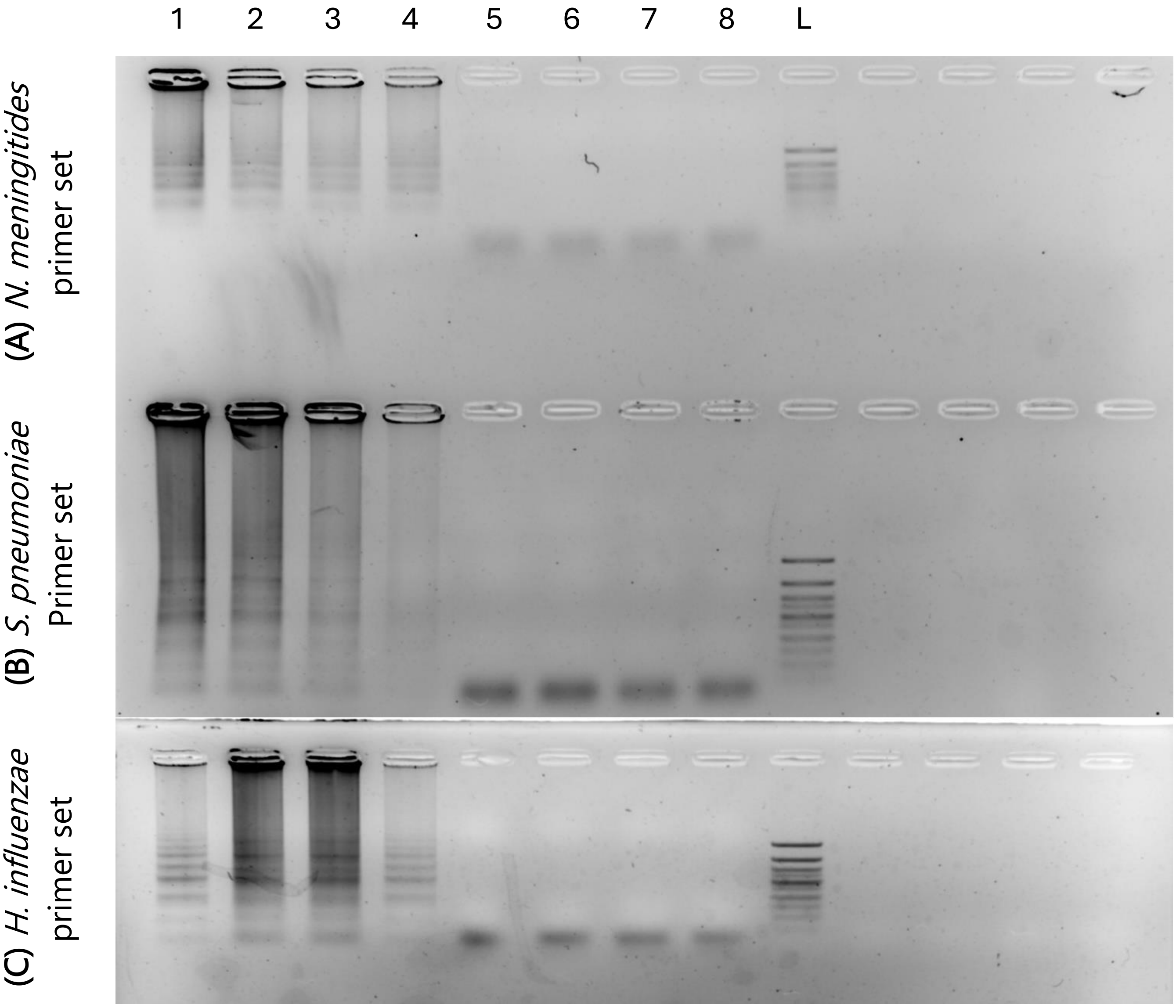


**Figure S1.** The limit of detection of the DAMP assay. Agarose 2% Gel electrophoresis of DAMP amplicons using **(A)** *N. meningitidis* primer set, **(B)** *S. pneumoniae* primer set, and **(C)** *H. influenzae* primer set. Reaction correspondind DNA in concentration **(1)** 100000 GE, **(2)** 10000 GE, **(3)** 1000 GE, **(4)** 100 GE, **(5)** 50 GE, **(6)** 1 GE, **(7)** 0 GE, **(8)** 10 GE, **(L)** 50bp+ DNA Marker.

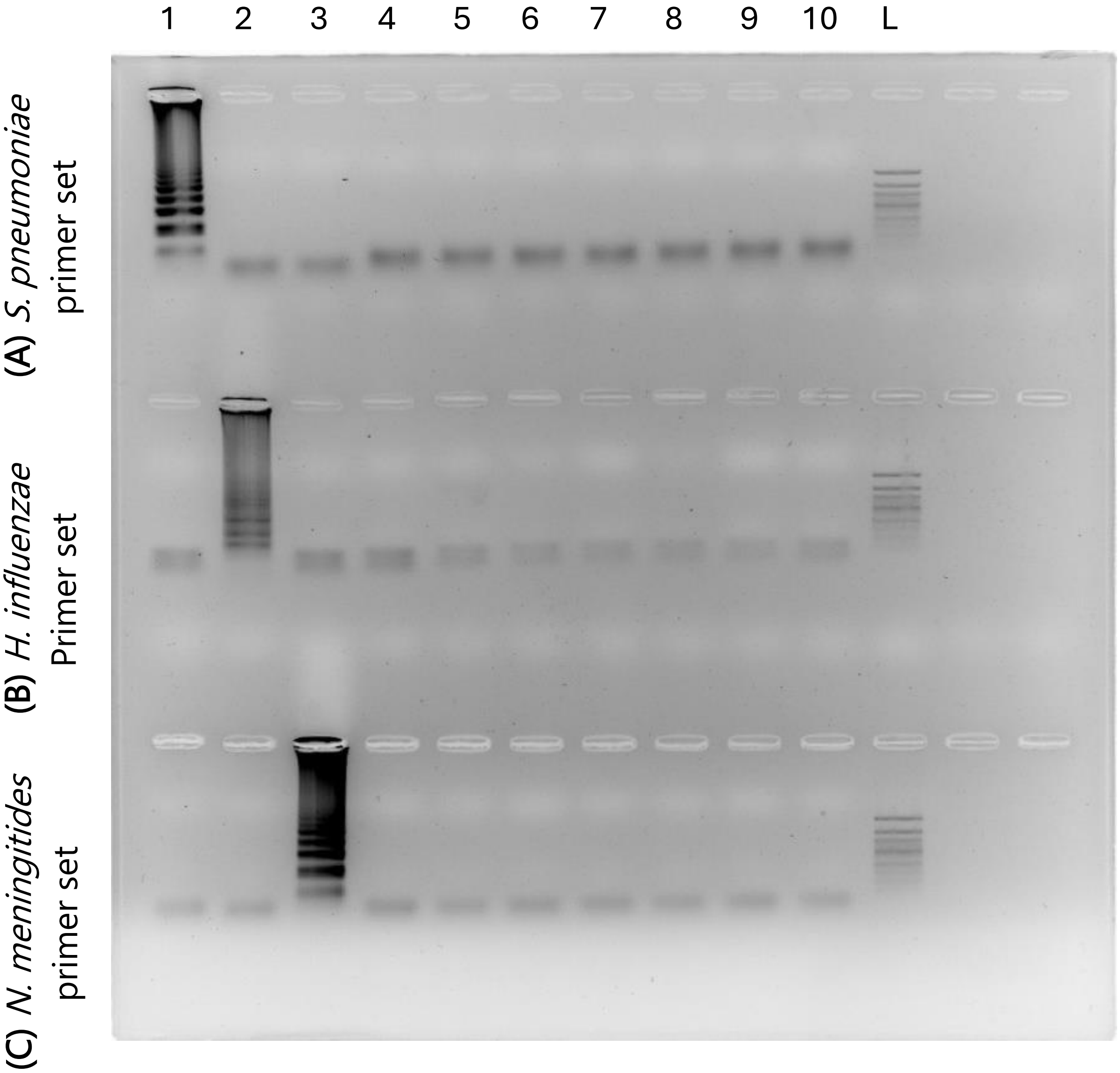


**Figure S2.** The cross-reaction of the DAMP assay. Agarose 2% Gel electrophoresis of DAMP amplicons using **(A)** *S. pneumoniae* primer set, **(B)** *H. influenzae* primer set, and **(C)** *N. meningitidis* primer set. Reaction with DNA of **(1)** *S. pneumoniae*, **(2)** *H. influenzae*, **(3)** *N. menengitidis*, **(4)** *S. agalacticae*, **(5)** *S. aureus*, **(6)** *L. monocytogenes*, **(7)** *K. pneumoniae*, **(8)** *A. baumannii*, **(9)** Human fragmented DNA, **(10)** Negative Control. **(L)** 50bp+ DNA Marker.

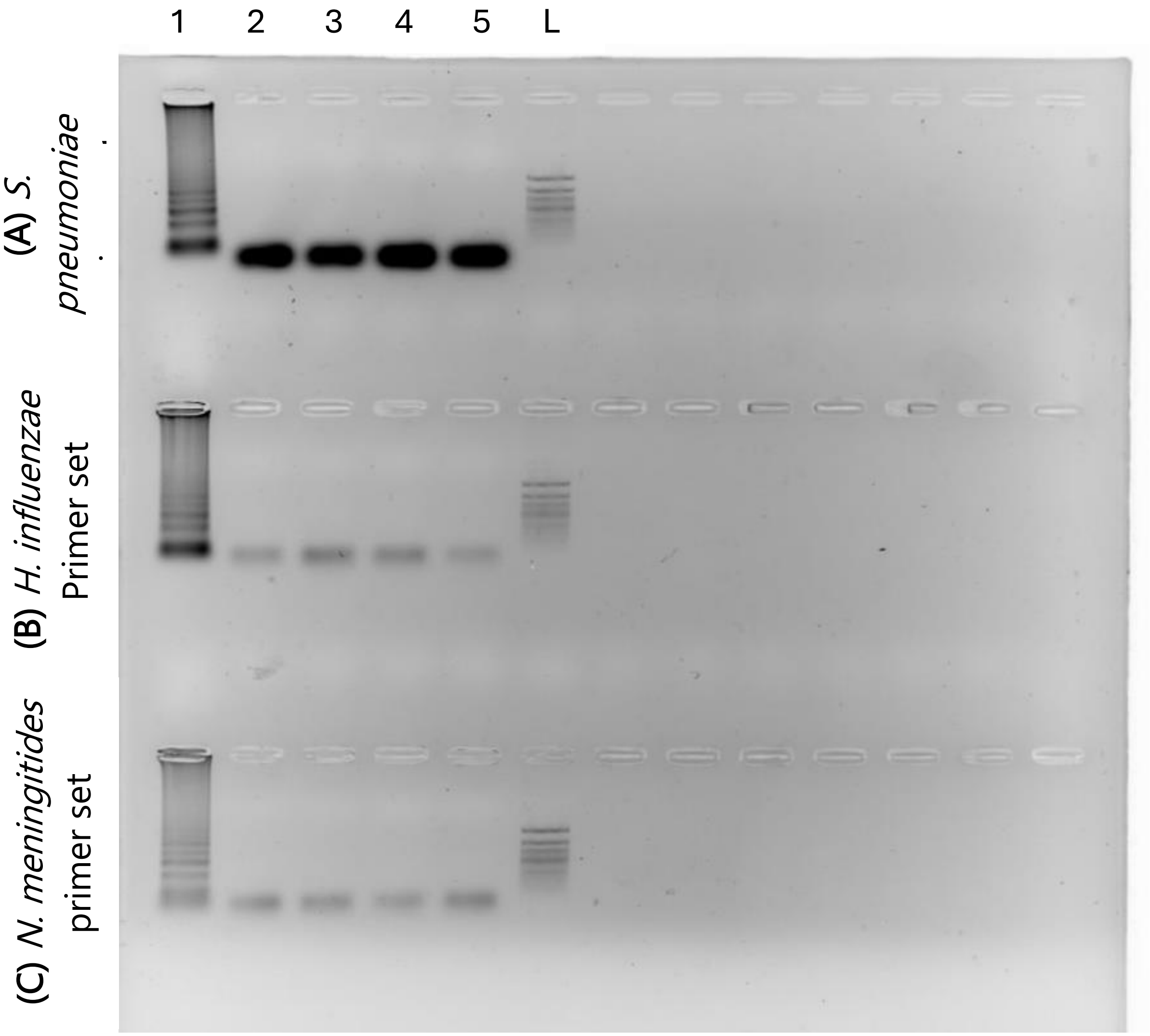


**Figure S3.** Testing of clinical samples by DAMP assay. Agarose 2% Gel electrophoresis of DAMP amplicons using **(A)** *S. pneumoniae* primer set, **(B)** *H. influenzae* primer set, and **(C)** *N. meningitidis* primer set. Reaction against the DNA exctracted from clinical samples conatining **(1)** Corresponding pathogen DNA, **(2)** *S. agalacticae*, **(3)** *S. aureus*, **(4)** *C. neoformans*, **(5)** non-infected CSF sample, **(L)** 50bp+ DNA Marker.
